# Development of heterotopic transplantation of the testis with the epididymis to evaluate an aspect of testicular immunology in rats

**DOI:** 10.1371/journal.pone.0177067

**Published:** 2017-05-05

**Authors:** Kai Yi, Naoyuki Hatayama, Shuichi Hirai, Ning Qu, Shogo Hayashi, Shinichi Kawata, Kenta Nagahori, Munekazu Naito, Masahiro Itoh

**Affiliations:** 1 Department of Anatomy, Tokyo Medical University, Tokyo, Japan; 2 Department of Anatomy, Aichi Medical University, Aichi, Japan; University of Hyderabad, INDIA

## Abstract

Transplantation of testicular cells and tissues has been studied for the investigation of immunology of the testis, which is an immunologically privileged organ. However, reports of transplant of the testis at organ level have been extremely limited because of technical difficulties of the orthotopic testis transplantation (OTT) in experimental animals. In the present study, we developed a new and simple model of the heterotopic testis transplantation (HTT), which is donor testis transplantation into the cervical region of recipients, in a syngeneic model in rats [donor Lewis (LEW) graft to LEW recipient]. The duration of HTT was significantly shorter and success rate higher than that of OTT. To histologically evaluate HTT, the local immune responses were compared among the syngeneic model, an acute rejection allogeneic model [donor Augustus Copenhagen Irish (ACI) graft to LEW recipient] and a chronic rejection allogeneic model (donor F344 graft to LEW recipient) at postoperative day 3. We found that allogeneic ACI grafts resulted in mild and not severe orchitic lesions, whereas immune responses of allogeneic F344 grafts seemed intact and were not significantly different from those of syngeneic LEW grafts. These results suggest that our new operative procedure will be useful in future for the investigation of the testicular immunology.

## Introduction

The testis is an immunologically unique site because new differentiation autoantigens-bearing spermatids and spermatozoa that appear in the seminiferous tubules long after the establishment of immune tolerance survive with no rejection by self-immunity under healthy conditions [1, 2]. It became evident that transplantation of testicular tissue fragments from newborn donors in several species (mouse, swine, chicken, ram, monkey, and goat) under the back skin of castrated “immunodeficient” mice resulted in the induction of spermatogenesis in donors to some degree [3–7]. However, from the aspects of testicular immunology, it is important to determine how grafts of testicular cells, tissues, or whole organ are immunologically accepted or rejected in “immunocompetent” recipients.

With regard to whole testis transplantation, Lee et al. were the first to formulate a model for orthotopic testis transplantation (OTT), which is characterized by an end-to-side anastomosis between the graft’s great arteriovenous segments with testicular arteriovenous vessels and the recipient’s great arteriovenous vessels that is subsequently retracted into the scrotum [8]. Several reports on modifications of Lee’s OTT model, including microsurgical techniques under 20–30× magnification and attaching the cuff of the donor’s common iliac vein with the testicular vein [9–12], have been published. However, the success rate for the OTT model was unstable because of technical difficulties, severe surgical invasion due to laparotomy, and long operative time. Consequently, there have been only six reports on the experimental application of OTT in rodents [8–13]. On the other hand, heterotopic transplantation techniques have been widely used for various organs, including the kidneys, heart, and small intestine, to investigate transplantation biology [14–16]. Heron et al. were the first to report heterotopic heart transplantation to the recipient’s cervical region using the cuff technique in rodents [17]. This operation technique has many benefits, such as complete anastomosis through ligation on only one occasion, less bleeding from anastomosis sites, shorter ischemic time of the graft, and improved success rate compared with orthotopic transplantation using the general suture technique. Recently, Nunes et al. attempted to transplant a whole testis into the greater omentum without vascular anastomosis in rats with resultant failure of graft survival [18]. Therefore, we performed heterotopic testis transplantation (HTT) with blood vessel anastomosis in the present study. The present study aimed to introduce a new and simple method of HTT for future elucidation of testicular immunology in rats.

## Materials and methods

### Animals

Male inbred Lewis rats (LEW/SsN Slc) at 10 weeks of age were used as the donors (n = 19), recipients (n = 31), and positive and negative controls (n = 24). Male inbred Fischer 344 rats (F344/NSlc, n = 6) and Augustus Copenhagen Irish rats (ACI/NSlc, n = 6) at 10 weeks of age were used as donors in the allogeneic models. The animals were purchased from SLC (Japan SLC, Shizuoka, Japan) and kept in the Laboratory Animal Center of Tokyo Medical University for 2 weeks prior to the experiments. Animals were maintained at 22°C–24°C and 50%–60% relative humidity with a 12 h light–dark cycle. This study was approved by the Tokyo Medical University Animal Committee (approval number: S-27003). All surgeries were performed under deep anesthesia using isoflurane, and all efforts were made to minimize suffering. Finally, rats were euthanized under general anesthesia with an isoflurane overdose (5%), which was maintained for 1 min after cessation of breathing and exsanguination during organ procurement.

### Study design

#### Experiment 1 (Ex. 1): Establishment of HTT in a syngeneic model

The operative time and success rate of the transplants between the HTT and OTT groups were evaluated. Vasectomy (Vx), ischemia (IS), and cryptorchidism (CR) groups were generated as positive controls for the consideration of possible effects of vas deferens occlusion, blood flow disturbance, and elevated temperature, respectively, during HTT. Therefore, this study was performed using the following six groups: the sham group as a negative control (n = 6), HTT group (n = 12), OTT group (n = 7), Vx group (n = 6), IS group (n = 6), and CR group (n = 6). For the OTT group, we used examples from Lee’s transplantation model where transplanted testes were retracted into the scrotal sac [8]. In the Vx group, the ipsilateral vas deferens was exposed without causing injury to adherent blood vessels, and the occluding ligature was applied to the vas deferens with a silk thread. The IS group received completely blocked blood flow to the ipsilateral testis by the arteriovenous ligation of the testis and vas deferens. In the CR group, the ipsilateral testis was translocated to the abdominal cavity after cutting the gubernaculum testis, and its epididymal adipose tissue was sewn to the peritoneum. In both HTT and OTT groups, the bilateral testes of recipients were castrated just after the transplantation. In the positive control groups (Vx, IS, and CR), the untreated contralateral testis was castrated. In the sham group, the unilateral testis was castrated. Criteria for transplantation success included pulsation of the testicular artery to the grafted testis and bleeding from the taken testis by macroscopic observation at postoperative day 3 when positive controls definitely exhibit spermatogenic disturbance and retention of degenerated germ cells in the seminiferous tubules and the epididymal ducts, respectively. The sampled testes and epididymides were histologically and immunohistochemically evaluated.

#### Experiment 2 (Ex. 2): Comparison of HTT in syngeneic and allogeneic models

We compared the local immune response in the transplanted testes in the syngeneic model (donor LEW graft to LEW recipient) with two allogeneic models (donor ACI graft to LEW recipient and donor F344 graft to LEW recipient) at postoperative day 3 after HTT. LEW rats receiving syngeneic grafts served as controls (LEW group). There are several reports that evaluate allograft rejection in various organs using LEW rats as recipients and ACI and F344 rats as donors [19, 20]. MHC haplotypes of these inbred rat strains have been established [21]. ACI rats exhibit major mismatched transplantation to LEW rats, which causes acute rejection, whereas F344 rats exhibit minor mismatched transplantation to LEW rats and does not cause acute rejection [22]. Therefore, allogeneic grafts taken from ACI (ACI group, n = 6) and F344 (F344 group, n = 6) rats were used as acute and chronic rejection models, respectively. The sampled testes and epididymides were histologically, immunohistochemically, and biochemically evaluated.

### Surgical procedure

Surgical procedures were performed under 20–30× magnification using a desktop operating microscope (SZ61, Olympus, Japan, Tokyo). The microsurgical instruments for this study included straight super-grip forceps, micro-hemostat clamps, and micro-dissecting scissors. All intraoperative rats were placed on a heating plate (35–40°C, Heater mat KN-475, Natsume Seisakusho Ltd., Tokyo, Japan) to account for decreased body temperature caused by anesthesia. All HTT, OTT, Vx, IS, CR, and sham operations were performed by two researchers who are experts at the experimental transplantation of hearts and kidneys in rodents.

### Donor preparation

**Fig 1:**
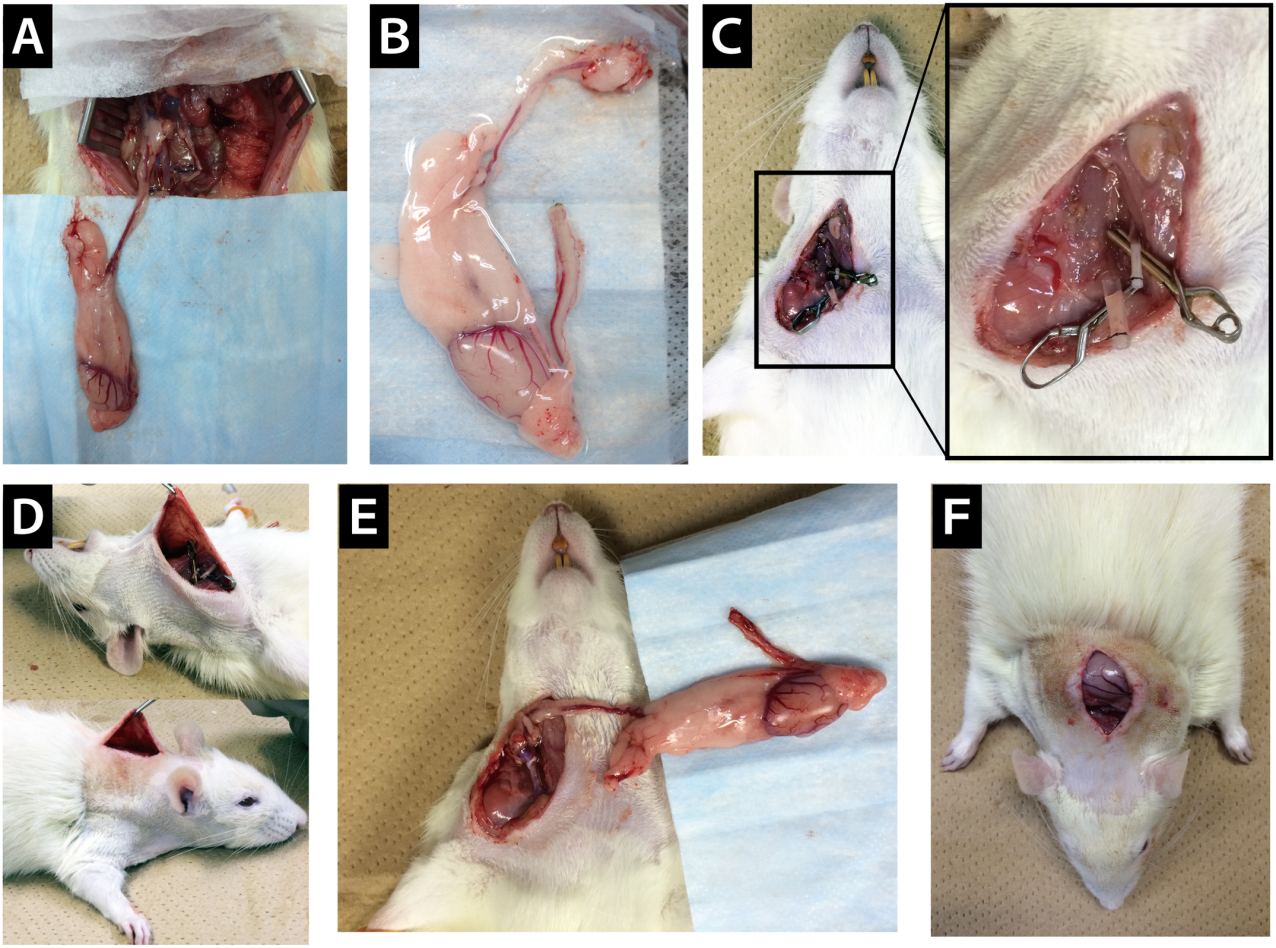
Surgical procedures for cervical heterotopic testis transplantation (HTT). A) The exposed testis with the epididymis and a part of the vas deferens for harvest in a donor rat. B) The harvested organs with nutrient vessels. C) Two cuffs were attached to the recipient’s common carotid artery and external jugular vein. D) The desquamated recipient’s skin for folding the transplanted testis. E) The reperfusion after cervical HTT in a recipient rat. F) The placement of the testis into the subcutaneous dorsal region of the neck in a recipient rat.

**Fig 2:**
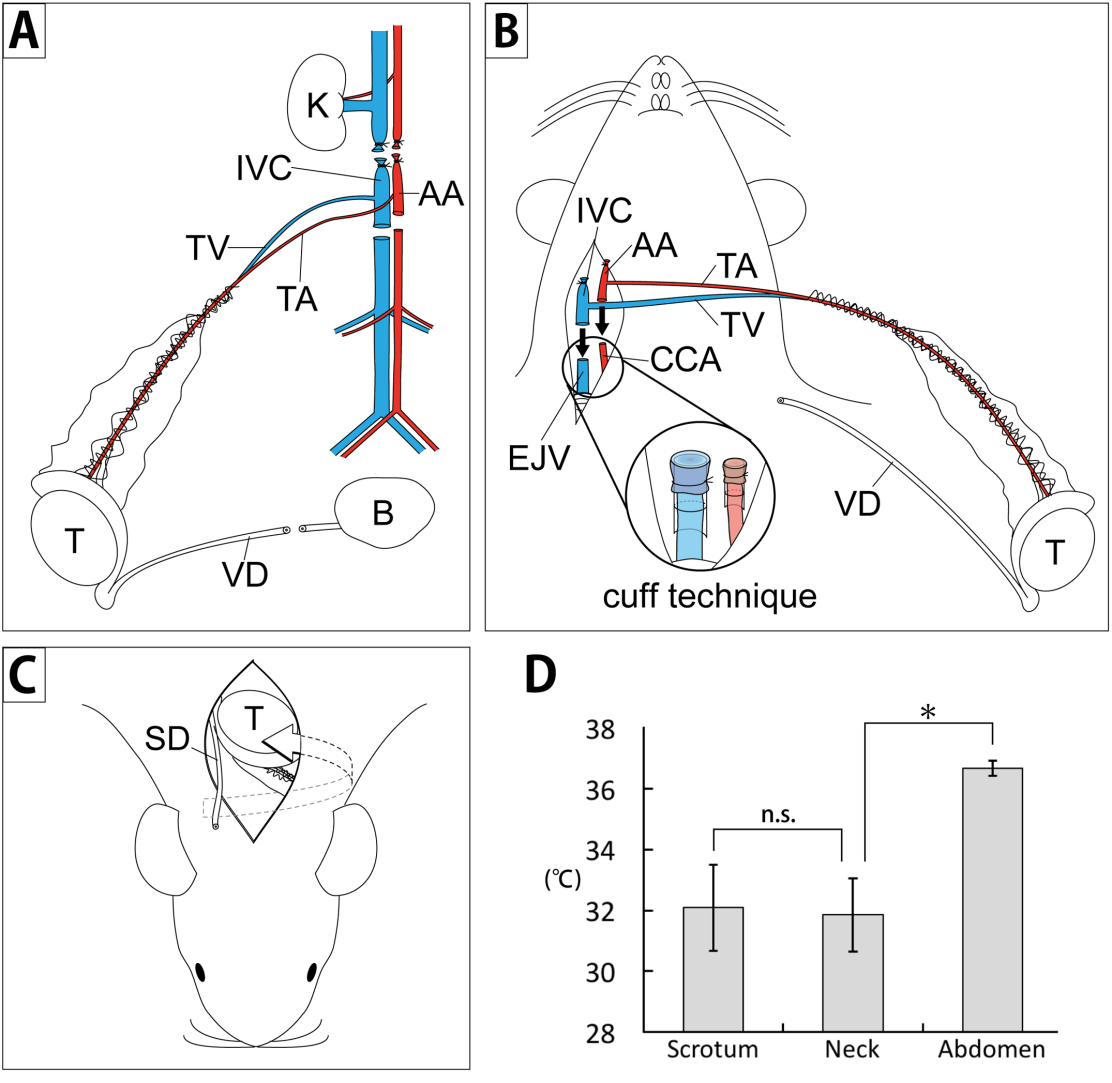
Schematic overview of cervical heterotopic testis transplantation (HTT) and subcutaneous temperature. A) The technique for harvesting the testis with the epididymis and a part of the vas deferens from a donor rat. B) The technique for cervical HTT with the cuff technique in a recipient rat. C) The fitting of the testis into the subcutaneous dorsal region of the neck and draining of the vas deferens outside the body in a recipient rat. D) The subcutaneous temperatures of rats were measured in the scrotal (32.1 ± 1.4°C, n = 6), cervical (31.9 ± 1.2°C, n = 6), and abdominal cavities (36.7 ± 0.2°C, n = 6). AA: abdominal aorta, B: bladder, CCA: common carotid artery, EJV: external jugular vein, K: kidney, VD: vas deferens, IVC: inferior vena cava, T: testis, TA: testicular artery, TV: testicular vein; n.s.: not significant, *P < 0.05.

For HTT, the animals were anesthetized using isoflurane (induction: 5%, maintenance: 1.5%–2%). The abdomen was shaved, swabbed with 70% alcohol, and opened through a long midline incision. Rats in which the right testicular artery arose from the abdominal aorta (AA) and the ipsilateral testicular vein arose from the inferior vena cava (IVC) were considered as suitable donors. IVC was ligated just above the entrance of the right testicular vein and transected 1 cm below the entrance of the right testicular vein. Similarly, AA was ligated just above the entrance of the right testicular artery and transected 1 cm below the entrance of the right testicular artery. The right vas deferens was transected near the bladder (Figs 1A and 2A). Prior to tissue removal of the testis with the epididymis and a part of the vas deferens, heparin (1000 IU/mL) was administered to reduce the risk of thrombosis. The entire graft was placed in a Petri dish with ice-cold saline (Fig 1B), and the delicate vascular pedicle (7–10 cm in length) was carefully examined under 20× magnification to make sure the twists were present (S1 Video).

For OTT, the procedure was based on the model established by Lee and Goldstein [8, 10]. Briefly, AA and IVC segments with the testicular arteriovenous vessels were obtained by a sharp division of the AA and IVC immediately below the clamp and tie. The testis with the epididymis and a part of the vas deferens was removed by cutting the gubernaculum and dividing the vas deferens.

### Recipient preparation

The procedure for HTT was based on the heterotopic heart transplantation method used by Heron et al. with some modifications [17]. The animals were anesthetized using isoflurane. The recipient was placed in a supine position with the head facing the operator. A 2 cm-long longitudinal incision was made from the submaxilla to the sternum. The right external jugular vein (EJV) was dissected and mobilized. The right common carotid artery (CCA) was exposed and mobilized as far as possible, without causing the transection of the sternocleidomastoid muscle. The proximal portion of CCA and EJV were then occluded with micro-hemostat clamps. The distal portion of CCA was ligated with 7–0 silk, and the right EJV was ligated with 7–0 silk at the level of the joint vein of the inframandibular/submandibular gland. The vessels were then divided between the clamps and distal ties, and the proximal cut ends were irrigated with heparinized saline. The right CCA and EJV were then passed through a Teflon cuff (SP tubing SP45; ID 0.58 mm × OD: 0.96 mm, SP95; ID: 1.2 mm × OD: 1.7 mm, Natsume Seisakusho Ltd., Tokyo, Japan) with superfine-tip forceps. By pulling the divided CCA and EJV with forceps, the proximal ends of the CCA and EJV were everted over the cuff and fixed using a circumferential ligature of 7–0 silk (Figs 1C and 2B). The recipient skin around the neck was pinched from the muscles and dorsal skin in the neck, and a 2 cm long longitudinal incision was made for folding of the transplanted testis (Figs 1D and 2C, S1 Video).

The procedure for OTT was based on the methods used by Lee and Goldstein [8, 10]. Briefly, the abdomen was entered via a midline incision. The intestines were retracted to the right, and AA and IVC were exposed and clamped.

### Transplantation

For HTT, the donor testis with the epididymis and a part of the vas deferens was transplanted into the neck of the recipient. AA and IVC with the testicular vessels of the donor were overlaid at the end of CCA and EJV with the cuff of the recipients and then fixed to each cuff with a circular ligature of 7–0 silk (Fig 2B). The clamp at the distal side of EJV was released first. CCA of the recipient was released soon after. The transplants were judged to be technically successful if the grafted testis returned to normal color, and active bleeding from the cut end of the vas deferens was observed at the completion of the procedure (Fig 1E). The transplanted testis was fitted to the posterior subcutaneous dorsal region of the neck, and the distal tip of the vas deferens was drained outside the body (Figs 1F and 2C). The cervical skin was closed with silk sutures (S1 Video).

The procedure for OTT was based on the methods used by Lee and Goldstein [8, 10]. Briefly, an end-to-side anastomosis was made between the graft’s great anteriovenous segments with the testicular vessels and the recipient’s great anteriovenous vessels using running sutures. This process was followed by reperfusion to the transplanted testis. The end-to-end anastomosis of the vasa deferentia of the graft and recipient was made using an interrupted suture. The testis was retracted into the scrotum, and the abdominal wound was closed. In both HTT and OTT groups, the bilateral testes of the recipient origin were castrated just after the transplantation. In the control groups (Vx, IS, and CR), the untreated contralateral testis was castrated. In the sham group, the unilateral testis was castrated.

### Subcutaneous temperature measurement

The subcutaneous temperature in the scrotum, neck, and abdominal cavity of the recipients was measured using a thermometer (PowerLab, ML312 temperature probe; ADInstruments Ltd., Australia) immediately before the removal of transplanted organs. Three temperature probes were inserted into each region under anesthesia. Temperatures were measured when the rectal temperature reached 36.5–37.0°C after regaining consciousness.

### Histopathological assessment

The isolated testes were fixed in Bouin’s solution and then embedded in plastic (Technovit 7100; Kulzer & Co., Wehrheim, Germany) without cutting the organs to prevent artificial damage to the testicular tissue. Sections (3–4-μm-thick) were obtained at 15–20-μm intervals, stained with Gill No. 3 hematoxylin and 2% eosin Y, and observed using a light microscope (BX51; Olympus, Shinjuku, Japan). The degree of spermatogenic disturbance was determined using Johnsen’s scoring system, which ranged from a score of 1 (no cells in the seminiferous tubules) to 10 (complete spermatogenesis) [23].

### Evaluation of Ki67-, CD4-, and CD8-positive cells in the testis

The isolated testes were frozen in liquid nitrogen and stored at −80°C until use. Cryosections (5 μm) were fixed in 1% glutaraldehyde for 10 min and then digested in pepsin solution (0.006% in 0.01 N HCl) for 10 min at 37°C. Samples were treated with 4 N HCl for 30 min at room temperature and then neutralized with borate buffer (0.1 M, pH 8.5). The samples were incubated with primary antibodies against rabbit anti-Ki67 (clone: ab16667, ×500; Abcam, Cambridge, UK), mouse anti-rat CD4 (clone: W3/25, ×200; AbD Serotec, Oxford, UK), and CD8 (clone: OX-8, ×200; Acris Antibodies GmbH, Herford, DE). Next, the samples were incubated with an alkaline phosphatase-labeled goat anti-rat IgG (Sigma-Aldrich). Immunoreactive cells were visualized using a Vectastain ABC Kit (Vector Laboratories) with 0.05% 3,3′-diaminobenzidine (DAB, Nickel Solution) and 0.01% H_2_O_2_ as the chromogen. As a semi-quantitative analysis, the number of seminiferous tubules with Ki67-positive cells per 100 round and oval-shaped seminiferous tubules was determined in each testis. The number of infiltrating CD4^+^ and CD8^+^ lymphocytes per mm^2^ of testicular interstitium was evaluated.

### Real-time RT-PCR

Total RNA was isolated from the transplanted testicular tissues using a TRIzol RNA extraction kit (Invitrogen, Carlsbad, CA, USA) and then reverse-transcribed into cDNA using a High-Capacity cDNA Archive Kit (Applied Biosystems, Foster City, LA, USA) according to the manufacturer’s instructions. The quantification of cDNA was performed using SYBR Premix Ex Taq II (TaKaRa, Ohtsu, Japan) and the Thermal Cycler Dice Real-Time System TP800 (TaKaRa). Glyceraldehyde-3-phosphate (GAPDH) was used as a housekeeping gene to normalize mRNA levels. The relative expression of real-time PCR products was determined using the ΔΔCt method to compare the mRNA expression of the target gene and GAPDH. Primers used in this study are listed in S1 Table.

### Statistical analysis

Values are presented as the mean ± standard deviations. The significance of differences was determined using either a paired unmatched t-test or an analysis of variance (ANOVA) with Tukey–Kramer post-hoc tests using Prism 6.0d for Macintosh (GraphPad Software, Inc., CA, USA). All statistical tests were performed as two-sided analyses, and P < 0.05 was used as the threshold for significance.

## Results

### Ex. 1: Establishment of HTT in the syngeneic model

**Fig 3:**
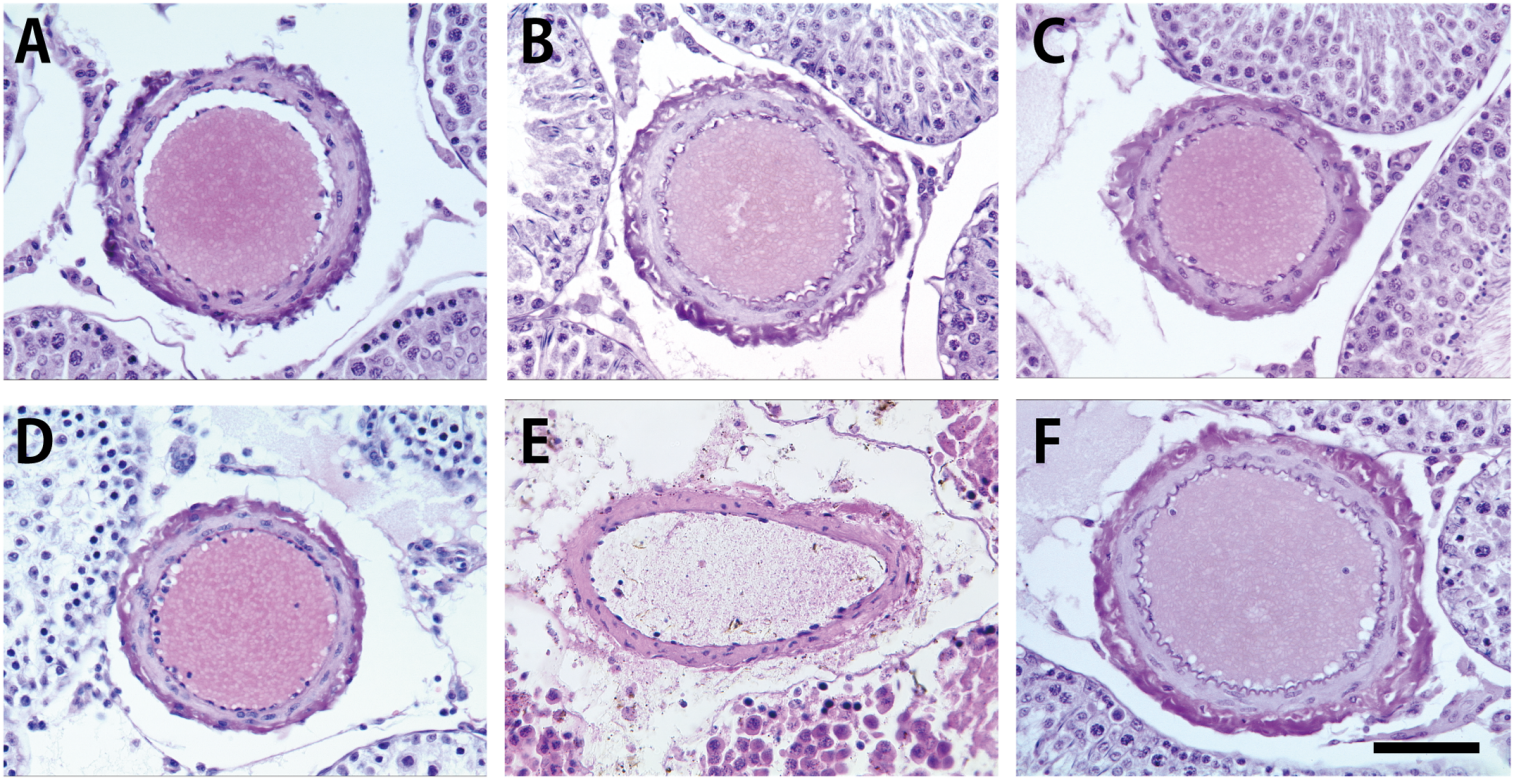
Histological feature of the arterioles inside the testis. A, sham group. B, heterotopic testis transplantation (HTT) group. C, orthotopic testis transplantation (OTT) group. D, vasectomy (Vx) group. E, ischemia (IS) group. F, cryptorchidism (CR) group. The arterioles of the HTT, OTT, Vx, and CR groups exhibited a normal appearance, including numerous red blood cells, that was similar to the sham group. In the IS group, only eosinophilic materials were observed inside the arteriole, and the vascular wall displayed degeneration. Black bar: 100 μm.

Histological examination of the arterioles inside the testis revealed that the sham, HTT, OTT, Vx, and CR groups were normal, including numerous red blood cells. However, inside the arterioles of the IS group, red blood cells were hardly observed and only eosinophilic materials were observed (Fig 3).

The operative time and success rate were compared between the HTT and OTT groups (Table 1). HTT was performed in 12 rats with a success rate of 100% (12/12) and a total operative time of 59.9 ± 7.1 min. The operative time for HTT was significantly shorter and the success rate was significantly higher than that for OTT, which had a total operative time of 154 ± 23.2 min and a success rate of 71% (5/7). The remaining two testes (2/7) underwent necrosis because of possible technical failures, resulting in nutrient vessel occlusion, as was observed in the IS group.

**Table 1:** Operative time and success rate.

Event	HTT	OTT
	Average time (min)	
Donor testis harvesting	27.1 ± 3.1*	58.2 ± 10.1
Recipient preparation	20.6 ± 2.8*	50.4 ± 5.3
Transplantation	12.2 ± 1.2*	45.4 ± 7.8
Graft ischemia time	25.2 ± 1.7*	92 ± 10.3
Total operative time	59.9 ± 7.1*	154.0 ± 23.2
Successful surgery	Success rate (%)	
	12/12 (100)*	5/7 (71)

HTT: heterotopic testis transplantation, OTT: Orthotopic testis transplantation

* P < 0.05 v.s. OTT model.

**Fig 4:**
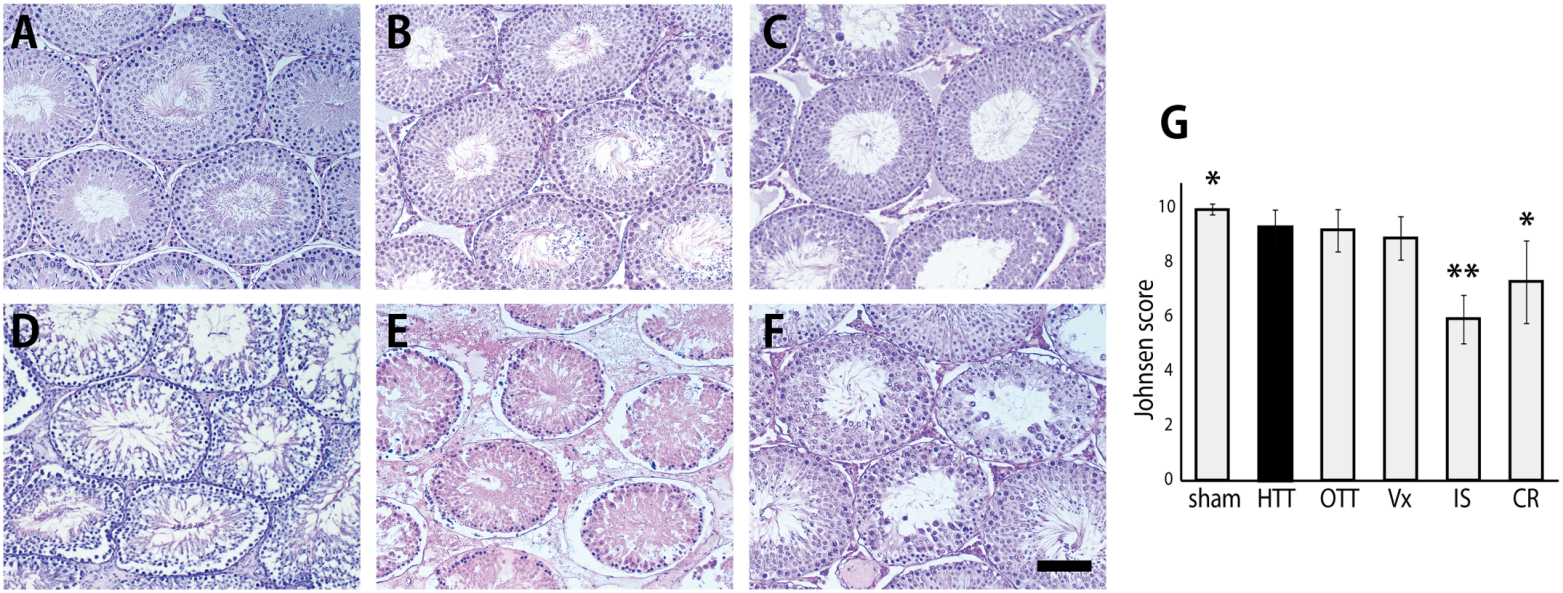
Histological sections of the testes. A, sham group (n = 6). B, heterotopic testis transplantation (HTT) group (n = 6). C, orthotopic testis transplantation (OTT) group (n = 6). D, vasectomy (Vx) group (n = 6). E, ischemia (IS) group (n = 6). F, cryptorchidism (CR) group (n = 6). G, Johnsen’s score for spermatogenesis. *P < 0.05 vs. HTT group. **P < 0.01 vs. HTT group. Black bar: 100 μm.

**Fig 5:**
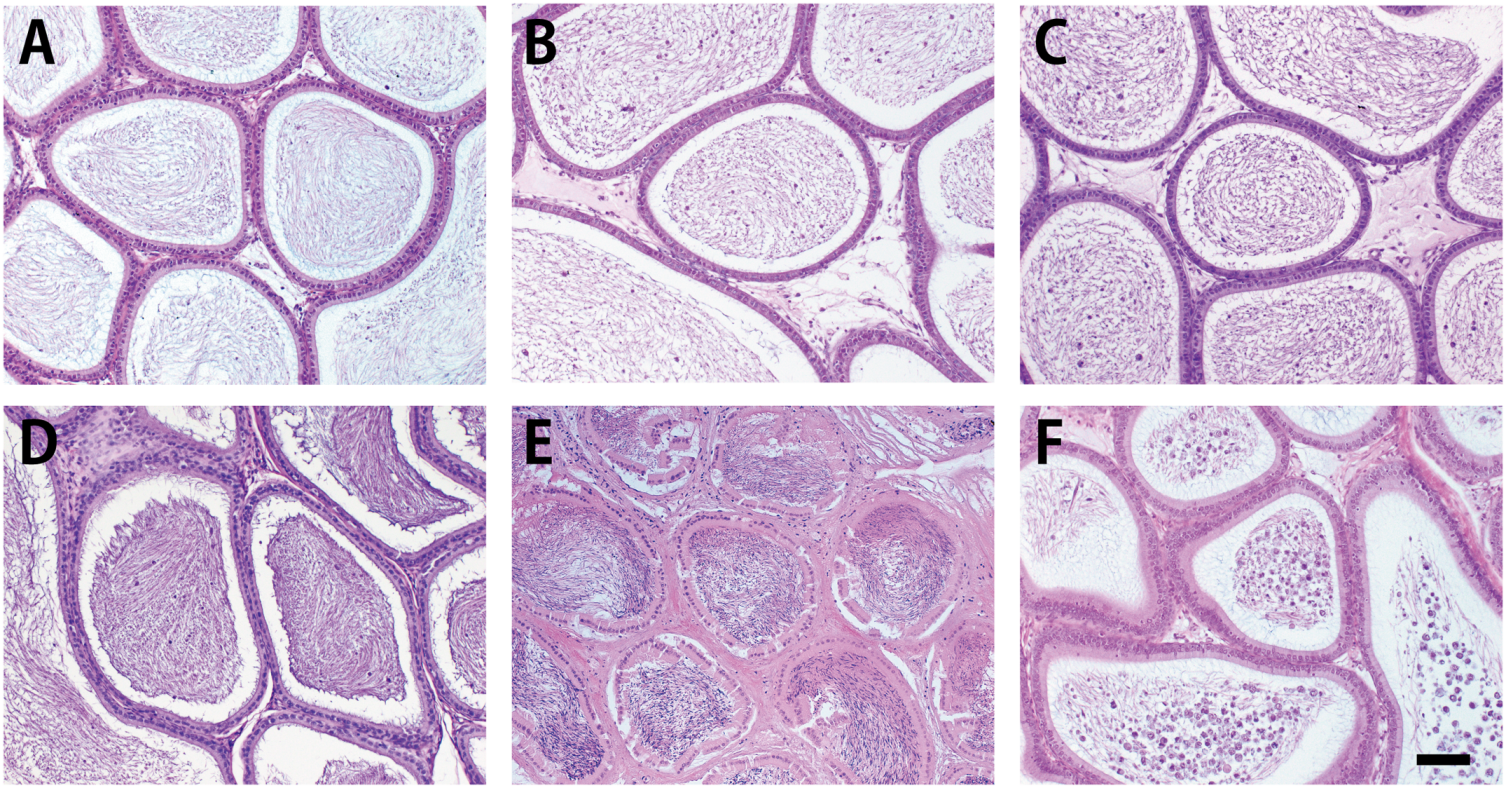
Histological sections of the epididymis. A, sham group. B, heterotopic testis transplantation (HTT) group. C, orthotopic testis transplantation (OTT) group. D, vasectomy (Vx) group. E, ischemia (IS) group. F, cryptorchidism (CR) group. The HTT and OTT groups had normal epididymal ducts and intra-duct compartments. The Vx group showed many retained spermatozoa in the epididymal ducts. The IS group showed coagulation necrosis of the epididymal ducts and adjacent interstitium. The CR group showed many deformed germ cells and absence of spermatozoa in the epididymal ducts. Black bar: 100 μm.

The histological appearance of the testes and the epididymides in HTT and OTT models were similar to those in the sham group. Most seminiferous tubules appeared normal and showed active spermatogenesis, whereas some seminiferous tubules exhibited disorganized spermatogenesis (Fig 4A and 4B). In the Vx group, almost all seminiferous tubules appeared normal, whereas the epididymal ducts were packed with more retained spermatozoa compared with those in the sham, HTT, and OTT groups (Figs 4D and 5D). The IS group exhibited ischemic atrophy and necrosis of the seminiferous tubules and epididymal ducts (Figs 4E and 5E). In the CR group, the elongated spermatids and spermatozoa were not found, and only immature round cells remained in the seminiferous tubules and epididymal ducts (Figs 4F and 5F).

**Fig 6:**
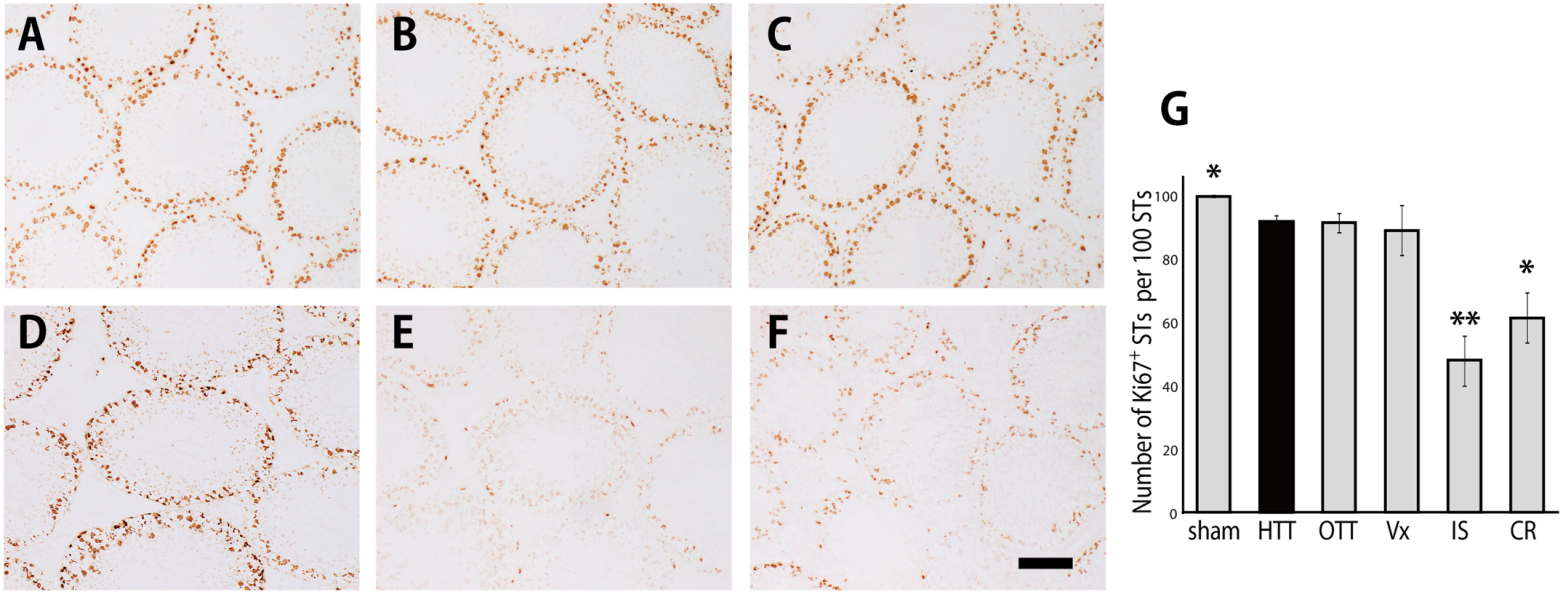
Ki67 immunohistochemistry of the testes. A, sham group (n = 6). B, heterotopic testis transplantation (HTT) group (n = 6). C, orthotopic testis transplantation (OTT) group (n = 6). D, vasectomy (Vx) group (n = 6). E, ischemia (IS) group (n = 6). F, cryptorchidism (CR) group (n = 6). G, the number of seminiferous tubules with Ki67-positive cells per 100 round and oval-shaped seminiferous tubules was determined in each testis. In all groups, Ki67-positive germ cells were observed along the basement membrane of the seminiferous tubules. STs: seminiferous tubules. *P < 0.05 vs. HTT group. **P < 0.01 vs. HTT group. Black bar: 100 μm.

Immunohistochemically, Ki67-positive germ cells (spermatogonia) were observed along the basement membrane of the seminiferous tubules (Fig 6). In the HTT group, the number of Ki67-positive germ cells was only slightly, but significantly, lower than that in the sham group (Fig 6A, 6B and 6G). In the OTT and Vx groups, no significant difference was detected compared with the HTT group (Fig 6B, 6C, 6D and 6G). In the IS and CR groups, the number of Ki67-positive germ cells was apparently reduced compared with that in the HTT group (Fig 6E, 6F and 6G).

### Ex. 2: Comparison of HTT in syngeneic and allogeneic models

**Fig 7:**
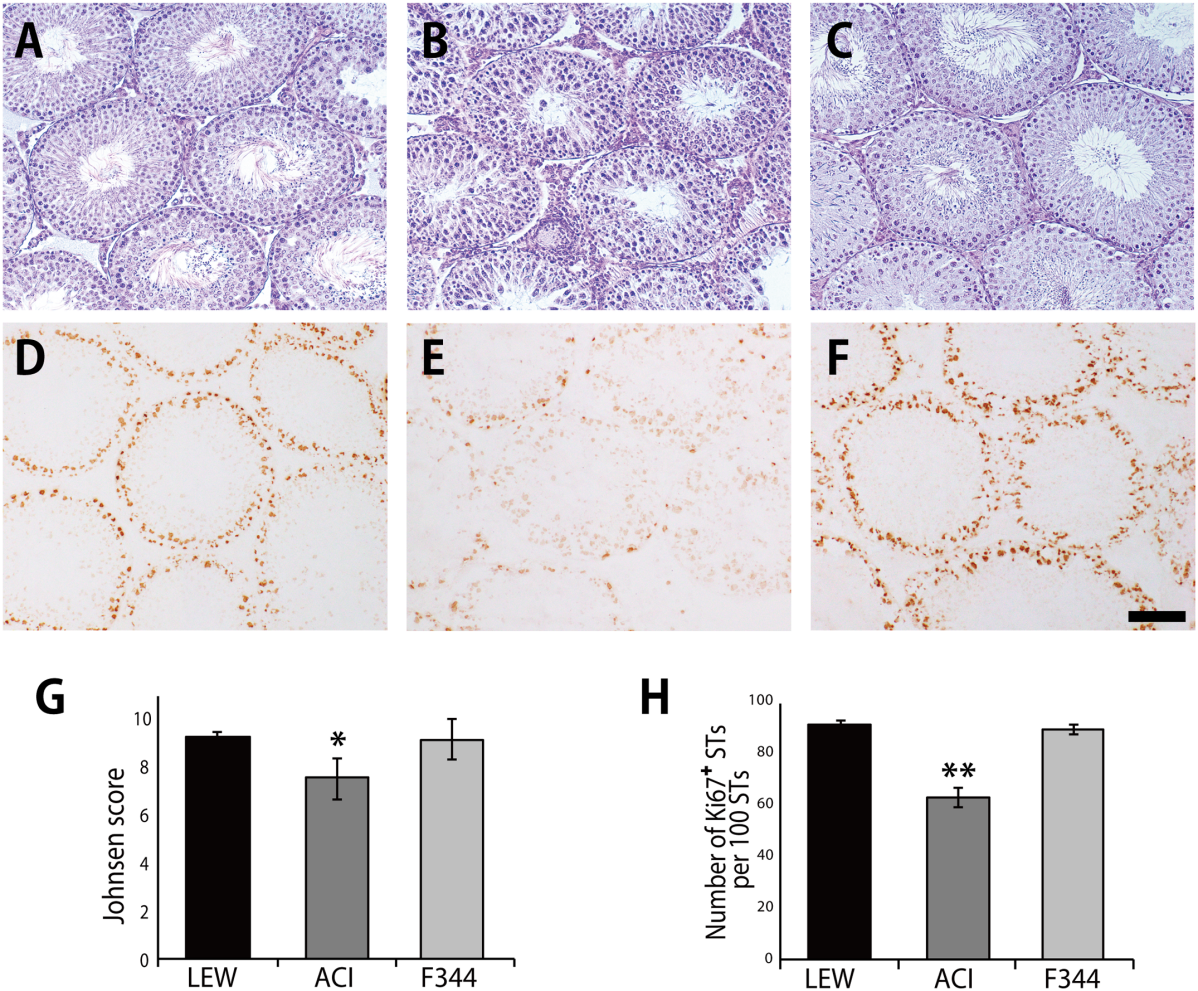
Histological and Ki67 immunohistochemical sections of the grafted testes. The LEW (A, D), ACI (B, E), and F344 (C, F) groups (n = 6). Johnsen’s score for spermatogenesis (G). The number of seminiferous tubules with Ki67-positive cells per 100 round and oval-shaped seminiferous tubules was determined in each testis (H). The score and number of Ki67-positive cells in the ACI group significantly decreased compared with that in the LEW group. STs: seminiferous tubules. *P < 0.05 vs. LEW group. **P < 0.01 vs. LEW group. Black bar: 100 μm.

**Fig 8:**
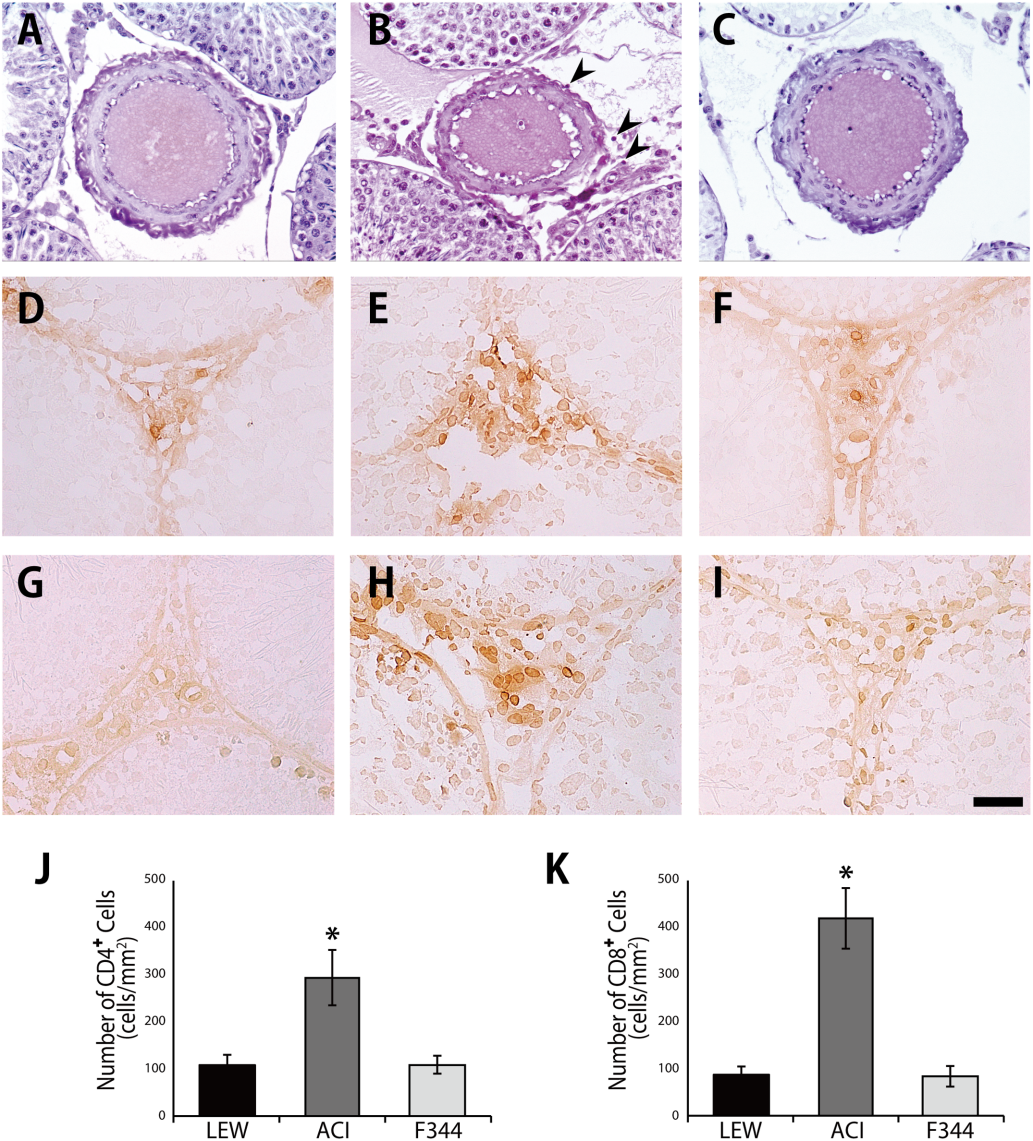
Histological and immunohistochemical sections of arterioles of the grafted testes. CD4 and CD8 immunohistochemistry in the grafts of the LEW (A, D, G), ACI (B, E, H), and F344 (C, F, I) groups. Histopathological changes were evaluated by counting the number of infiltrating CD4^+^ and CD8^+^ cells per mm^2^ of the testicular interstitium (J, K). Injuries to the vascular endothelium and significant infiltration of CD4^+^ and CD8^+^ lymphocytes around the arterioles were found in the ACI group. The arrow heads indicate lymphocytes. *P < 0.01 vs. LEW groups. Black bar: 50 μm.

The histological appearance and Ki67-staining pattern of the grafts in the F344 group were not significantly different from those in the LEW group (Fig 7A, 7C, 7D, 7F, 7G and 7H). In contrast, a notable spermatogenic disturbance was observed in the grafts of the ACI group (Fig 7B, 7E, 7G and 7H). Injuries to the vascular endothelium and lymphocytic infiltration around the capillaries were found in the grafts of the ACI group (Fig 8B); in addition, many CD4^+^ T cells and CD8^+^ T cells were detected (Fig 8E and 8H). A smaller number of CD4^+^ T and CD8^+^ T cells were observed in the grafts of the LEW and F344 groups (Fig 8A, 8F, 8G, 8I, 8J and 8K).

**Fig 9:**
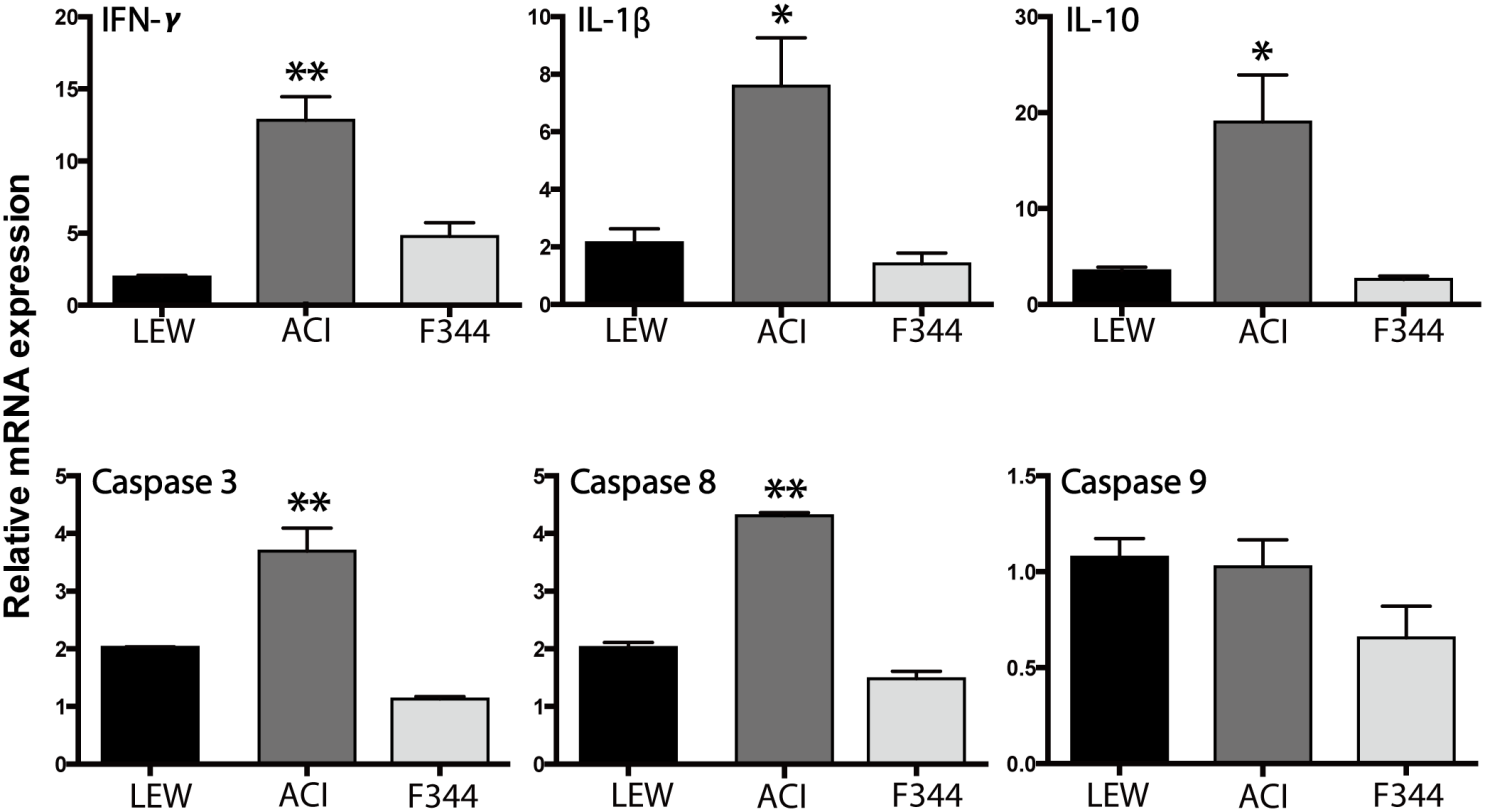
mRNA levels in the grafted testes detected by real time RT-PCR. In the ACI group, mRNA expression levels of inflammatory mediators, such as IFN-γ, IL-1β, and IL-10, and apoptosis-related genes, such as caspase 3 and caspase 8, were significantly higher than in the LEW group (n = 3 for each group). *P < 0.05 vs. LEW group. **P < 0.01 vs. LEW group.

Real-time RT-PCR analyses revealed that mRNA expression levels of inflammatory cytokines (IFN-γ, IL-1β, and IL-10) prominently increased in the grafts of the ACI group compared with those of the LEW group (Fig 9). The expression levels of caspase 3 and caspase 8 (apoptosis-related genes), but not caspase 9, were significantly higher in the ACI group than in the LEW group. In contrast, these cytokines remained unchanged in the grafts of the F344 group.

## Discussion

This study was performed to assess testis transplantation into a heterotopic area (cervical region) with blood vessel anastomosis in rats using a new HTT technique. Compared with the OTT model, the HTT model had a significantly shorter operative time and improved success rate. Furthermore, the testes transplanted by HTT appeared histologically intact, as in the sham group, in the syngeneic model (donor LEW graft to LEW recipient). In contrast, transplanted testes in the acute rejection model (donor ACI graft to LEW recipient) exhibited significant inflammatory changes compared with those in the chronic rejection model (donor F344 graft to LEW recipient) and syngeneic model.

The operative time for the OTT model was previously reported as 3–5 h [10–12]. Similar results were obtained in our OTT model, which took approximately 2.5 h (Table 1). One of the reasons for such a long operative time is that the OTT model requires anastomosis of the very thin and narrow vessels of the testis. The testicular artery and vein for OTT are approximately 0.2 and 0.8 mm in diameter, respectively. In contrast, the HTT model affirmed its use as an experimental transplantation model with a high success rate and short operative time of approximately 1 h (Table 1), thus improving the efficiency of this experimental discipline. AA and IVC of the donors for HTT are approximately 1 and 1.5 mm in diameter, respectively. In general, ischemia of the testis is harmful to spermatogenesis [24, 25]. The short operative time (25.2 ± 1.7 min in HTT; Table 1) contributes to a reduction in ischemia of the donor testis, and this technique is minimally invasive for the recipient rats. The average ischemic time of our OTT experiment was 92 ± 10 min (Table 1). Previous reports on the ischemic time for OTT models ranged from 35 to 110 min [8, 9, 11, 12, 24]. In this study, there were no histological differences between the HTT and OTT groups. However, it is possible that more testicular damage may be induced in OTT than in HTT in a long-term follow-up after transplantation. Another advantage of HTT is that it is less invasive to the recipient. Only a 2-cm-incision on the neck was required for HTT, whereas OTT requires a laparotomy.

The following three issues must be considered in HTT. First, the effect of the testicular temperature at the cervical lesion should be assessed. It is well known that the process of spermatogenesis is highly sensitive to variations in the environment, particularly fluctuations in temperature. One of the most widely used models to investigate the effect of temperature on the testis is the experimental cryptorchid model. In previous reports, testicular weight decreased 4 to 14 days after the experimental induction of cryptorchidism [26, 27]. In addition, the diameter of seminiferous tubules reduced, and only a few tubules showed active spermatogenesis in a rat model [26, 27]. In our study, the subcutaneous temperature of the neck was significantly lower than that of the abdominal cavity, and there was no significant difference in the local temperature between the scrotum and cervical subdermal space (Fig 2D). The CR group showed spermatogenic disturbances, as evidenced by Johnsen scores and Ki67 immunostaining. These disturbances were not observed in the HTT group (Figs 4 and 6). These results suggest that HTT is not affected by cervical subcutaneous temperature.

The second issue relates to blood flow disturbance caused by folding of the long and thin testicular arteriovenous vessels into the cervical subcutaneous region. Testicular torsion results in obvious spermatogenic disturbances because the blood supply to the testis is cut-off by the twisting of the testis [28]. Several investigators examined effects of the short-term ligation of the testicular artery. In warm ischemic testes, the testis weight and sperm count significantly decreased after 1–5 h, and the number of *in situ* terminal deoxynucleotidyl transferase dUTP nick-end labeling-positive (measurement of cell death) germ cells and the volume and density of intravascular polymorphonuclear leukocytes increased compared with those in naïve testis [24, 25, 29]. Ischemic necrosis and obvious spermatogenic disturbances were histologically and immunohistochemically observed in the IS group at postoperative day 3 (Figs 4E and 6E). In contrast, the HTT group showed definite blood flow at postoperative day 3, as evidenced by the pulsation of the testicular artery to the grafted testis and bleeding from the testis. The histological features of our HTT model also supported the evidence of no or little blood flow disturbance (Fig 3).

The third issue is the effect of the draining of the donor vas deferens to the outside of the recipient body for HTT. There is a possibility that the distal tip of the vas deferens is dried and occluded. A previous study described epididymal distension resulting from raised intraluminal pressure through the occlusion of spermatozoa in the early phases of a vasectomy [30]. In the late phase, a spermatic granuloma is formed and an anti-sperm autoantibody is produced in rats [31]. In the Vx group, there was no evidence of significantly disorganized seminiferous tubules, but there were many retained spermatozoa in the epididymal ducts at postoperative day 3 (Fig 5D). However, in the HTT group, no such retention of spermatozoa was observed. These results indicate that spermatozoa are continuously drained from the epididymal duct to the distal tip of the vas deferens, and thus redundant spermatozoa are degenerated and then absorbed with no back pressure to the epidydimal duct.

In general, the testis is an immunologically suppressed organ, i.e., an immunologically hospitable site in which allogeneic tissues, such as the thyroid gland and pancreatic islets, show long-term survival [32–35]. The blood–testis barrier, formed by tight junctions of Sertoli cells, partitions the interstitial blood compartment of the testis from the adluminal compartment of seminiferous tubules [36]. The testicular interstitium outside the blood–testis barrier, where many resident macrophages are normally present, is also protected from attack by the body’s immune system. It became evident that not only Sertoli cells but also Leydig cells, testicular macrophages, peritubular myoid cells, and blood vessels endothelia are involved in testicular immune privilege, and these testicular cells express and secrete numerous immunoregulatory molecules, including androgens, macrophage migration inhibitory factor, activin, Fas ligand, protein S, and immunosuppressive cytokines, such as IL-10 and transforming growth factor-β, which play critical roles in regulating immune responses in the testes [36–38]. In particular, endothelial barrier antigens are expressed in microvessels of the testis and brain but not in those of other organs including the heart, liver, intestine, adrenal gland, skeletal muscle, thymus, lymph node, pancreas, thyroid, skin and pituitary gland [39, 40]. Therefore, we expected that transplanted testes of the ACI group would not be promptly rejected at postoperative day 3 in the present study. However, the results showed that histological injuries to the vascular endothelium, infiltration of CD4^+^ and CD8^+^ lymphocytes around arterioles, and increased mRNA expression levels of IFN-γ, IL-1β, IL-10, caspase 3, and caspase 8 were found in the transplanted testes of ACI group but not in those of F344 group. This suggests that the testicular arterioles play little role in immune privilege and become targets for acute allograft rejection under the experimental condition of the present study. There remains a possibility that testicular arterioles equipped with endothelial barrier antigens relatively protect the testicular parenchyma from immune attack compared with those in other transplanted organs. To verify whether the severity of rejection of transplanted testis is milder than that of other transplanted organs in ACI group, we are currently comparing local immune responses in the transplanted testes with those in other transplants, such as the heart and kidney from postoperative day 1 to postoperative day 60. In this long-term evaluation, we will also observe chronic rejection model in F344 group to compare with the syngeneic model.

Chemotherapy or radiation therapy for malignant cancers often injure the spermatogenesis of young patients. In particular, prepubertal patients may suffer from permanent infertility because mature spermatozoa in semen cannot be cryopreserved prior to therapy. Thus, transplantation of children’s testes and epididymides to their fathers or brothers before receiving medical therapy may be helpful for obtaining mature spermatozoa from the transplanted testes at a future date. It is also possible that the transplanted testes in recipients are transplanted back into donors after medical treatment. Therefore, the establishment of the HTT model may contribute to this unmet clinical need.

## Conclusions

We developed a new practical method for the transplantation of the testis with the epididymis in rats. HTT is technically more simple and reproducible than OTT. This newly developed operative procedure could be useful for the further investigation of testicular immunology.

## Supporting information

S1 Table List of primers used in this study. (XLSX)

S1 Video Rat cervical heterotopic testis transplantation (HTT) surgery. This video illustrates each step of the HTT surgical procedure at sextuple speed with a running time of 3 min.
(MP4)
